# Cholera outbreak in a naïve rural community in Northern Nigeria: the importance of hand washing with soap, September 2010

**DOI:** 10.11604/pamj.2018.30.5.12768

**Published:** 2018-05-04

**Authors:** Saheed Gidado, Emmanuel Awosanya, Suleiman Haladu, Halimatu Bolatito Ayanleke, Suleman Idris, Ismaila Mamuda, Abdulaziz Mohammed, Charles Akataobi Michael, Ndadilnasiya Endie Waziri, Patrick Nguku

**Affiliations:** 1 Nigeria Field Epidemiology and Laboratory Training Programme, Abuja, Nigeria; 2 Department of Community Medicine, Ahmadu Bello University, Zaria, Nigeria; 3 Epidemiology Unit, Ministry of Health, Jigawa State, Nigeria

**Keywords:** Cholera, case-control study, hand washing, Northern Nigeria

## Abstract

**Introduction:**

Cholera outbreaks in rural communities are associated with high morbidity and mortality. Effective interventions to control these outbreaks require identification of source and risk factors for infection. In September, 2010 we investigated a cholera outbreak in Bashuri, a cholera naïve rural community in northern Nigeria to identify the risk factors and institute control measures.

**Methods:**

We conducted an unmatched case-control study. We defined a case as any resident of Bashuri community two years and above with acute watery diarrhea with or without vomiting and a control as any resident two years and above without acute watery diarrhea and vomiting. We recruited 80 hospital-based cases and 80 neighborhood controls. We collected and analyzed data on demographic characteristics, clinical information and risk factors. Laboratory analysis was performed on 10 stool samples and 14 open-well samples.

**Results:**

Mean age was 29 years (± 20 years) for cases and 32 years (± 16 years) for controls; 38 (47.5%) of cases and 60 (75%) of controls were males. Compared to controls, cases were less likely to have washed hands with soap before eating (age-adjusted odds ratio (AAOR) = 0.27, 95% confidence interval (CI): 0.10-0.72) and less likely to have washed hands with soap after using the toilet (AAOR = 0.34, 95% CI: 0.15-0.75). Vibrio cholerae O1 was isolated from six stool samples but not from any open-well samples.

**Conclusion:**

Unhygienic hand washing practices was the key risk factor in this outbreak. We educated the community on personal hygiene focusing on the importance of hand washing with soap.

## Introduction

Cholera is an acute bacterial enteric disease characterized by sudden onset of profuse, painless diarrhea and vomiting. It is caused by Vibrio cholerae serogroups O1 and O139. The disease is acquired through ingestion of contaminated food or water (faeco-oral route) and is transmitted through several mechanisms. The main reservoir of the disease are humans [1]. The incubation period ranges from a few hours to 5 days, usually 3 days [2]. Cholera may cause severe dehydration and death within a few hours. In many cholera outbreaks, at least 90% of the cases are mild or asymptomatic [3]. The case fatality rate (CFR) may exceed 50% in untreated patients; with proper and timely rehydration and treatment, CFR is usually less than 1%. Cholera remains a global threat to public health and has been described as one of the key indicators of social development [4]. Although, the disease is no longer an issue in countries where minimum environmental hygiene standards are met, it remains a threat in almost every developing country. The typical settings for cholera are rural and peri-urban slums where basic urban infrastructure is missing. In endemic countries, about 2.8 million cholera cases occur each year with average global annual incidence rate of two cases per 1000 at risk population [5]. In 2006, 236 896 cholera cases were reported to World Health Organization from 52 countries with 6311 deaths, representing an increase of 79% compared with the number of cases reported in 2005 [6, 7]. This increased number of cases is the result of several major outbreaks that occurred in countries where cases have not been reported for several years. In recent years, Nigeria has witnessed recurrent cholera outbreaks. According to surveillance data obtained from the Epidemiology Division, Federal Ministry of Health, between January 2004 and December 2008, outbreaks of cholera have been reported in 12 out of the 36 States in the country with 74,881 cases and 1,387 reported deaths (Unpublished data). Within the last two years, cholera outbreaks have been reported in Benue, Sokoto and Zamfara States, all in northern Nigeria. In September, 2010, an outbreak of cholera was reported in Bashuri community, a cholera naïve rural community in Jigawa State, north-west Nigeria. We investigated this outbreak to identify the source, determine the risk factors for contracting infection and implement appropriate control measures. This paper describes the epidemiological methods employed in the investigation, summarizes the key findings and highlights the public health actions undertaken to control the outbreak.

## Methods

**Study site and population**: The outbreak investigation was conducted in Bashuri, a rural community located about 19 kilometers South of Dutse, the Jigawa State capital in northern Nigeria. Bashuri is an agrarian community with an estimated population of 2,400 inhabitants. Majority of the inhabitants are Hausas and Islam is their main religion. The main sources of drinking water for most inhabitants are open wells scattered across the community. Toilet facility in most households is pit-latrine. Refuse are disposed by burning. The community has never experienced any outbreak of cholera and/or gastro-enteritis in the past. There is no health facility in this community.

**Descriptive methods**: In this outbreak, suspected cholera cases presented to the cholera treatment camp set up in the community. We adapted the integrated disease surveillance and response (IDSR) recommended case definition for cholera to identify suspected cases. We defined a suspected cholera case as any resident of Bashuri, two years old and above, with acute watery diarrhea (three or more motions in 24 hours) with or without vomiting from 3^rd^ September, 2010 [8]. We interviewed and physically examined some cases at the treatment center to verify diagnosis and ensure that they met the case definition. We developed a line-list to collect information from all suspected cases. We searched for additional suspected cases in the community and patent medicine vendors. We analyzed the line-list data to characterize the outbreak in time, place and person and to develop a plausible hypothesis for cholera transmission in the community.

**Analytical methods, case-control study**: We conducted an unmatched case-control study to determine factors associated with infection. A case was defined as any person 2 years old and above residing in Bashuri community and presenting with acute watery diarrhea with or without vomiting, while a control was defined as any person age 2 years or more residing in Bashuri community without acute watery diarrhea and vomiting. We enrolled 80 cases and 80 controls to identify an odds ratio of 3 (for a risk factor on which intervention would have a significant impact), assuming 20% prevalence of exposure among control with 95% confidence interval and power of 80% [9]. The sample size was determined using the Statcal function of Epi-Info Software [10]. We sourced and recruited the cases consecutively from among the patients that presented at the treatment camp. The controls were sourced from the community; each control was selected from the 3^rd^ homestead to the right of the household of a case. We used structured questionnaires to collect data on demographic characteristics, exposures and associated factors from both cases and controls, and clinical information from the cases only.

**Laboratory methods**: We collected 10 stool specimens from suspected cases within 4 days of onset of symptoms and before commencement of antibiotics. The specimens were transported in Cary Blair transport medium to a tertiary health facility in the State where laboratory analysis was performed using thiosulfate-citrate-bile-sucrose (TCBS) agar to culture *vibrio* organism and polyvalent antisera to determine the serotypes. Fourteen water specimens collected from selected open wells were analyzed for *Vibrio* organism and *Escherichia coli (E. coli)* at the State Rural Water Board Laboratory.

**Data management**: We entered data into Epi-Info statistical software and performed univariate analysis to obtain frequencies and proportions, and bivariate analysis to obtain odds ratios and determine associations, setting p-value of 0.05 as the cut-off for statistical significance. We also performed unconditional logistic regression to adjust for possible confounders and identify the independent factors for contracting cholera infection. Factors that were significant at p < 0.05 in the bivariate analysis and biological plausible variable such as age and sex were included in the model. We tested for collinearity among predictors using the Chi square test for binomial variables. The goodness of fit of the model was tested using the Pearson goodness of fit test. In the final model, only variables that were found to significantly affect the outcome at P < 0.05 were retained. Data management was done exclusively using Epi-info software (version 4.3.2).

**Ethical consideration**: The study was conducted as part of an outbreak response which is one of the mandates of the Ministry of Health. Permission to conduct the study was granted by the Jigawa State Ministry of Health and the district head of Bashuri community. Informed consent was obtained from all subjects interviewed. Confidentiality of all the subjects was assured and maintained during and after the study.

## Results

**Figure 1:**
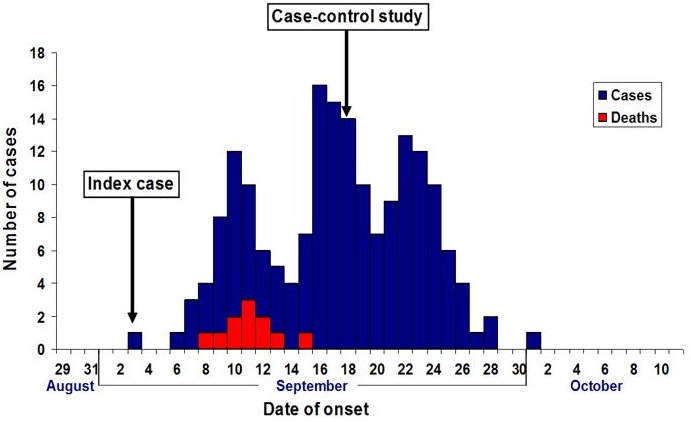
Epidemic curve of suspected cholera outbreak in Bashuri, Jigawa State, September-October, 2010

**Figure 2:**
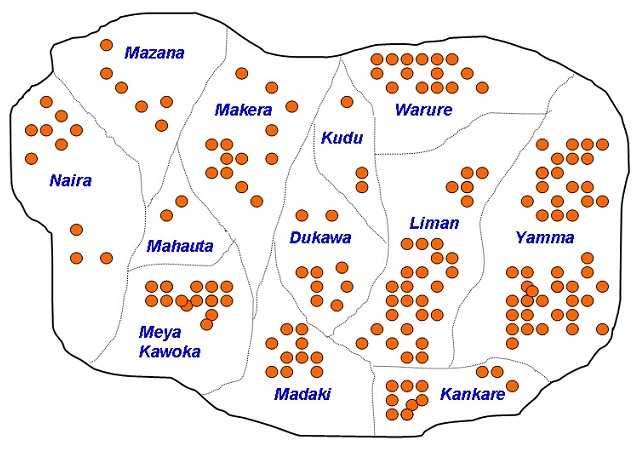
Distribution of suspected cholera cases by Angwas (hamlets), Bashuri community, Dutse LGA, Jigawa State; September-October, 2010

**Descriptive Epidemiology**: A total of 181 cases with 11 deaths (CFR: 6%) were recorded, 95 (52.5%) were males. Cumulatively, the highest number of cases was recorded in the age group 30 years and above in both sexes. However, age group 0-4 years had the highest attack rate (Table 1). Figure 1 shows the epidemic curve of the outbreak. The index case, and the primary case in this outbreak, developed symptoms on 3^rd^September 2009. The epidemic curve has a propagated pattern with three peaks, and a period of about 5 to 6 days between the peaks. The outbreak spanned over a period of one month, with most deaths recorded in the early phase. Figure 2 shows the spot map for the outbreak. There was widespread distribution of cases in the community. One hundred and five (58%) of the cases were managed as in-patients.

**Table 1:** Age specific attack rate of cholera cases, Bashuri community, Jigawa State, September 2010

Age group (in years)	Number of cases	Percentage of total population	Estimated age group population	Age specific Attack rate/100 population
0 – 4	45	20.0%	480	9.4
5 – 14	26	27.6%	662	3.9
15 – 29	48	21.4%	514	9.3
30 and above	62	31.0%	744	8.3
**Total**	181	100	^+^2, 400	7.5

+ Estimated total population of Bashuri community is 2,400

**The index case**: The index case was a 25-year old male farmer. On 31^st^ August, 2009, he travelled to Mubi in Adamawa state, north-east Nigeria, where confirmed cholera outbreak was ongoing. While in Mubi, he freely interacted and ate with members of his host household. While going back to Bashuri on 3^rd^ September, 2009, he developed diarrhea and vomiting and was treated as out-patient at a health facility close to his community. The vomiting subsided after receiving treatment; however, diarrhea persisted for some days while in Bashuri community. His mother and some other household members who took care of him developed same symptoms after about 4 days following his full recovery.

**Case-control study**: The mean age was 29 years (± 20 year) for cases and 32 years (± 16 years) for controls. The male gender constituted 47.5% of the cases and 75% of the controls. Seventy-nine (99%) of cases and 75 (94%) of controls possess at most primary education. Sixty-five (81%) of cases and 68 (85%) of controls are farmers. All (100%) of the cases and controls use well water for drinking and other domestic purposes. Eighty four percent of the cases and 93% of the controls respectively utilize pit latrine as the method of human waste disposal. In the one-week preceding the onset of their illness, 8 (10%) of the cases and 9 (11%) of the controls had travelled out of Bashuri community. All (100%) of the cases and controls practice Islam. Persons who washed their hands with soap and water before eating were significantly less likely to contract infection than those who did not (OR = 0.23, 95% CI: 0.09-0.60). Similarly, persons who washed their hands with soap and water after using toilet were significantly less likely to contract infection than those who did not (OR = 0.27, 95% CI: 0.12-0.58). Additionally, contact with a person with diarrhea was a significant risk factor for infection (OR = 2.40, 95% CI: 1.18-4.88). However, water hygiene related factors and consumption of any of the common local delicacies was not significantly associated with infection (Table 2). The unconditional logistic regression shows that contact with a person with diarrhea remained as a significant risk factor (AAOR = 2.92, 95% CI: 1.28-6.67) while washing hand with soap and water before eating remained as a significant (AAOR = 0.24, 95% CI:0.08-0.72) protective factor (Table 3).

**Table 2:** Univariate analysis result showing association of exposure factors for cholera infection in Bashuri, Jigawa State, September 2010

Exposure factors	Cases n = 80	Controls n = 80	Odds Ratio (95% CI)	p-value
Washing hands with soap and water before eating	6 (7.5%)	21 (26.3%)	0.23 (0.09 – 0.60)	0.003
Washing hands with soap and water after using toilet	11 (13.8%)	30 (37.5%)	0.27 (0.12 – 0.58	0.001
Contact with a person with diarrhea	64 (80.0%)	50 (62.5%)	2.40 (1.18 – 4.88)	0.023
Not preparing water before drinking	74 (92.5%)	70 (87.5%)	1.76 (0.55 – 5.80)	0.43
Storing drinking water in a container without cover	78 (97.5%)	75 (93.8%)	2.60 (0.49 – 13.8)	0.22
Eating cold Alele	19 (23.8%)	16 (20.0%)	1.25 (0.59 – 2.64)	0.70
Eating cold Kosai	50 (62.5%)	43 (53.8%)	1.43 (0.76 – 2.69)	0.34
Drinking cold Kunnu	66 (82.5%)	62 (77.5%)	1.37 (0.63– 2.98)	0.55
Drinking Zobo	28 (35.0%)	32 (40.0%)	0.81 (0.43 – 1.53)	0.64
Eating cold Awara	12 (15.0%)	16 (20.0%)	0.71 (0.31 – 1.61	0.53
Eating cold fried fish	26 (32.5%)	33 (41.3%)	0.69 (0.36 – 1.31)	0.33
Eating cold Masa	17 (21.3%)	23 (28.8%)	0.67 (0.32 – 1.38)	0.36

**Table 3:** Result of unconditional logistic regression of risk factors for cholera infection in Bashuri, Jigawa State; September 2010

^+^Exposure Factors	Odds Ratio (95% CI)	p-value
Contact with a diarrhea case	2.92 (1.28-6.67)	0.01
Washing hands with soap before eating	0.24 (0.08-0.72)	0.01

+ Model includes age and gender

**Laboratory findings**: Six of the 10 stool samples analyzed yielded growth of *Vibrio cholera*, serogroup O1, all Ogawa serotype. All the 14 water samples yielded growth of *E.coli*, but none yielded growth of *Vibrio cholera*.

## Discussion

This paper describes the investigation of a cholera outbreak in a cholera-naïve rural community in northern Nigeria using both descriptive and analytical epidemiological methods. This community has never experienced any cholera outbreak in the past, despite the presence of favorable conditions for cholera transmission. The current outbreak most likely, resulted from a distortion of the agent-host-environment equilibrium following importation of the disease by the index case from an area with ongoing cholera transmission. This partly, underscores the need to identify and properly interview the index case during outbreak investigation to provide useful information necessary to fully understand outbreaks context and dynamics especially in rural areas. The epi-curve of the outbreak revealed a propagated epidemic pattern which probably indicates that the disease was transmitted from person to person [11, 12]. Person to person transmission of cholera has been documented in previous cholera outbreak investigation in Haiti, Zambia and other countries [13-15]. Although, this mode of transmission is not as common as the well-known common point-source transmission of the disease, it highlights the need to conduct appropriate epidemiological study to identify the sources of infection and risk factors for disease transmission during an outbreak. The results of the case-control study indicated that contact with a person with diarrhea was a significant risk factor for contracting infection, further strengthening the person-to-person transmission hypothesis generated from the descriptive epidemiology. Considering the nature and tradition of the extended family relationship in this setting and indeed, in most rural communities in northern Nigeria, this epidemiological description is most likely plausible.

Furthermore, the epi-curve revealed a new generation of cases after the case-control study with a peak occurring about four days after the study, and gradually tapering down till the end of the outbreak. This pattern could be explained by two phenomena. Firstly, based on the result of the case-control study, we instituted immediate public health actions including a community-wide dissemination of health messages focusing on hand washing with soap and water. We believed this could have probably influenced decision at the individual and community levels with more inhabitants practicing this personal hygiene. Intuitively, potential cases could have been averted with subsequent decline in cholera incidence. The cases that constituted the new generation were perhaps, those incubating the disease at the time of the case-control study. Secondly, this epidemiological pattern could represent a phenomenon reflecting the natural course of cholera infection [16]. Typically, with prompt case management, the case fatality rate in a cholera outbreak is less than 1% [3]. The case fatality rate recorded in this outbreak was 6%, with most of the deaths occurring at the early stages of the outbreak. This finding could be attributed to two factors. Firstly, for a disease that is alien to this setting, the community and local surveillance system might not have detected the initial cases early enough to permit prompt case management. Secondly, late arrival of State and National investigators due to delay in notification of the outbreak, could result in poor case management by the local responders at the early stage of the outbreak. The epi-curve indicated that, although cholera cases increased in propagated pattern as the outbreak progressed, the number of deaths declined considerably, with no death recorded after the first generation of cases.

We identified unhygienic hand washing practices as the key risk factor for cholera infection in this outbreak. This clearly demonstrates the importance of hand washing with soap and water in reducing the risk of contracting cholera infection. Several studies have shown the health benefits of hand washing with soap and water in the prevention and spread of communicable diseases [17-20]. Although, in the setting where this outbreak occurred, getting very clean water to wash the hands with soap might be difficult, studies have indicated that washing the hands with soap with either clean or dirty water is very effective in removing hand contamination and reduces the spread of infectious agents from person to person [18]. A major public health implication of this finding is the need to scale up health communication and community sensitization on the health benefits of hand washing with soap as a personal hygiene practice to reduce the incidence of infectious diseases spread by direct physical contact including cholera, influenza and viral hemorrhagic fevers among others [21-23]. The laboratory analysis identified *Vibrio cholera* in more than half of the stool samples, but in none of the 14 water samples tested. It is indeed, not impossible that well water, which constitute the major source of water for drinking and other domestic purposes in this community was not contaminated with *Vibrio cholerae*. This could partly, explain the lack of association between water related risk factors and disease transmission in this outbreak, further strengthening the person-to-person pattern of transmission demonstrated by the case-control study. However, negative culture of *Vibrio cholerae* in the well water samples does not absolutely indicate the absence of this organism. In certain circumstances, it is possible for the organisms to enter into a viable but nonculturable state wherein they may not form colonies on traditional bacteriological culture plates. In such state, cell densities may be extremely low and insufficient to permit detection of the organism [24, 25].

A major strength of this study was the use of both descriptive and analytical epidemiological methods in the outbreak investigation. With a combination of these methods, we identified a relatively uncommon mode of cholera transmission namely person-to-person transmission via contact, a reflection of the socio-cultural characteristics of inhabitants of the community. Identification of this key risk factor permitted the implementation of evidenced-based, simple intervention to control the outbreak. This is instructive considering that in most cholera outbreaks, investigators are biased towards water and food decontamination. The findings and conclusions of this outbreak investigation should be interpreted within the context of a major limitation we encountered during the case-control study. We did not test the stools from the control for *Vibrio cholerae* to determine their appropriate case status. Considering that in many cholera outbreaks, about 90% of cases are mild or asymptomatic [3], our study participants might have been possibly, misclassified.

## Conclusion

In conclusion, the cholera outbreak in this community was imported by the index case from an area with ongoing cholera transmission. Community spread was facilitated by contact with cases. The key risk factor for contracting infection was unhygienic hand washing practices. To control the outbreak, we instituted timely and appropriate case management including prompt administration of oral rehydration salts or intravenous fluids depending on the severity of cases. Additionally, we administered antibiotics to moderate and severe cases to shorten the duration of the diarrhea, reduce the volume of rehydration fluid needed and shorten the duration of the vibrio excretion [26]. Furthermore, we organized and conducted an intensive health education focusing on improved personal hygiene but with emphasis on the importance of hand washing with soap. We also educated the community members on food and water safety measures as well as improved environmental sanitation. We recommended to the Jigawa State Ministry of Health to strengthen surveillance for cholera in neighboring communities for early detection of possible spill-over of the outbreak, extend health education and public enlightenment activities on cholera to other communities and train relevant health workers on surveillance and case management of cholera.

### What is known about this topic

- Typical settings for cholera infection are rural and peri-urban slums where basic infrastructure like potable water and hygienic waste disposal facilities are lacking. In these settings, transmission occurs majorly by consumption of contaminated water or food;
- Case fatality rate during cholera outbreaks is determined by how early cases present to treatment facilities, swiftness of initiation of treatment and quality of case management. With excellent case management, case fatality rate should not exceed 1%.

### What this study adds

- In spite of the presence of favorable environmental conditions for cholera transmission in this community, no previous outbreak of cholera has been recorded in the community - Although, it is widely believed that poor environmental conditions, with indiscriminate human waste disposal and lack of potable water drive cholera transmission, this study indicates that these factors alone are not adequate for disease transmission–an external factor is required to distort the epidemiological equilibrium for disease transmission to occur. The study indicated that importation of the disease-causing agent from an external milieu was responsible for the outbreak in this hitherto naïve community;
- In typical cholera outbreaks, water samples from the outbreak epi-center is always contaminated with *Vibrio cholerae* fueling disease transmission via ingestion of food and water substances - This outbreak investigation demonstrated that water samples might remain uncontaminated during cholera outbreak especially in rural setting - This emphasizes the need for investigators to be meticulous particularly during cholera outbreak investigation and explore all possible perspectives in order to identify factors propagating the outbreak.

## Competing interests

The authors declare no competing interests.
